# Protein-Based Anchoring Methods for Nucleic Acid Detection in Lateral Flow Format Assays

**DOI:** 10.3390/mi14101936

**Published:** 2023-10-16

**Authors:** Kira Hallerbach, Khadijeh Khederlou, Lael Wentland, Lana Senten, Steven Brentano, Brian Keefe, Elain Fu

**Affiliations:** 1 School of Chemical, Biological, and Environmental Engineering, Oregon State University, Corvallis, OR 97331, USA; 2 HP Inc., Palo Alto, CA 94304, USA; 3 HP Inc., San Diego, CA 92127, USA

**Keywords:** nucleic acid detection, lateral flow assay, high-density capture, nitrocellulose substrate, protein anchor, streptavidin–biotin linkage

## Abstract

The use of lateral flow assays to detect nucleic acid targets has many applications including point-of-care diagnostics, environmental monitoring, and food safety. A sandwich format, similar to that in protein immunoassays, is often used to capture the target nucleic acid sequence with an immobilized complementary strand anchored to a substrate, and then to visualize this event using a complementary label nucleic acid bound to a nanoparticle label. A critical component of high-sensitivity nucleic acid detection is to utilize high-density capture surfaces for the effective capture of target nucleic acid. Multiple methods have been reported, including the use of streptavidin-based protein anchors that can be adsorbed to the lateral flow substrate and that can utilize the high-affinity streptavidin–biotin linkage to bind biotinylated nucleic acid capture sequences for subsequent target nucleic acid binding. However, these protein anchors have not been systematically characterized for use in the context of nucleic acid detection. In this work, we characterize several protein-based anchors on nitrocellulose for (i) capturing the robustness of the attachment of the protein anchor, (ii) capturing nucleic acid density, and (iii) targeting nucleic acid capture. Further, we demonstrate the signal gains in target nucleic acid hybridization made by increasing the density of capture nucleic acid on a nitrocellulose substrate using multiple applications of protein loading onto nitrocellulose. Finally, we use our high-density capture surfaces to demonstrate high-sensitivity nucleic acid detection in a lateral flow assay (in the context of a SARS-CoV-2 sequence), achieving a LOD of approximately 0.2 nM.

## 1. Introduction

Nucleic acid detection in a lateral flow format is widely used in research and in commercially available tests [1]. The application areas of interest range from point-of-care diagnostics to environmental monitoring and food safety [2]. A target nucleic acid sequence is captured via a sandwich format, analogous to that for protein immunoassays. Specifically, the target nucleic acid sequence is linked via nucleic acid hybridization to the substrate-bound capture nucleic acid, and to the label nucleic acid sequence that is bound to a nanoparticle label for signal generation. A strong motivation for nucleic acid detection in a lateral flow format is the promise of lower limits of detection from upstream nucleic acid amplification, as well as the advantages of high specificity and strain identification that are enabled by targeting nucleic acid sequences in a point-of-care assay format. A critical requirement in the development of effective nucleic acid detection in lateral flows is the fabrication of a reproducible, high-density layer of the capture nucleic acid sequence on the nitrocellulose substrate.

Nitrocellulose is a natural choice of lateral flow substrate [3], due to its well-characterized flow properties, tortuous structure that affords a high surface-area-to-volume ratio for the attachment of capture species, and commercial availability. Further, a well-known characteristic of nitrocellulose is that it adsorbs antibodies well [3]. However, for DNA targets, nitrocellulose has a lower adsorption affinity, particularly to DNA fragments of less than 500 bp (whereas alternative membranes such as nylon can bind even smaller fragments of DNA well) [4]. Thus, there have been reports of various strategies to attach DNA capture sequences to nitrocellulose for DNA detection in the lateral flow format. These can be divided into two broad categories, one that uses protein anchors and one that directly attaches the DNA capture sequences to nitrocellulose using salt [5], UV light [6,7,8], and/or heat [5,9]. For the protein anchor strategy, the most common methods involve the use of a biotin-binding protein that is attached to (i) a biotinylated capture DNA sequence of interest via the high-affinity streptavidin–biotin linkage and (ii) a nitrocellulose substrate via the nonspecific adsorption of the protein onto nitrocellulose. Specifically, streptavidin (SA) has been used successfully to achieve nucleic acid detection in lateral flows [10,11] (and there is a history of SA use on nitrocellulose for biotinylated protein attachment). However, SA is also known to be susceptible to rinsing off nitrocellulose substrates [12,13], especially when the rinse fluid contains a surfactant such as Tween 20 (often used to mitigate nonspecific adsorption in the context of gold nanoparticle labels). Another biotin-binding protein that has been used as a protein anchor for biotinylated nucleic acids (and biotinylated proteins) is a polymer of streptavidin, Polystreptavidin R (pSA) [14,15]. A third biotin-binding protein that has been used in the context of biotinylated protein binding is a nitrocellulose-binding streptavidin protein (NC-SA) [12,13]. NC-SA is a recombinant form of SA which exhibits an additional NC binding functionality, but exists in monomeric form. Finally, anti-biotin antibodies have also been reported as a protein anchor for use in nucleic acid and protein detection assays [16,17]. Although these different species have been used as protein anchors in assay development, there has been little characterization of them and no direct comparison of them in the context of nucleic acid detection that we are aware of.

**Figure 1:**
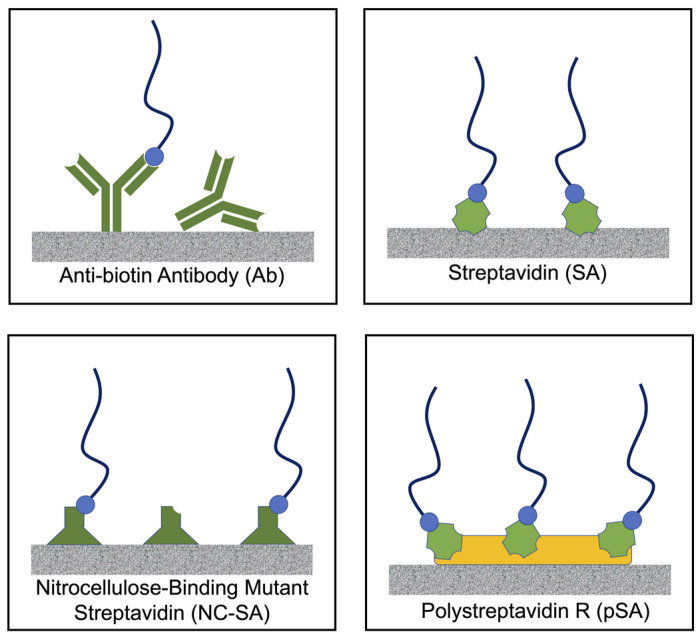
Schematic of protein-based DNA anchoring methods on nitrocellulose. These proteins, anti-biotin IgG antibody (**top left**), streptavidin (**top right**), nitrocellulose-binding streptavidin (**bottom left**), and Polystreptavidin R (**bottom right**), bind biotin, and can enable the tethering of biotinylated nucleic acid sequences that are complementary to the target nucleic acid for effective capture.

In the current report, we consider common protein anchoring systems in the context of nucleic acid detection (Figure 1), comparing the protein anchor robustness of adsorption to nitrocellulose and the density of capture nucleic acids. We further characterize the increase in the level of protein adsorbed onto nitrocellulose after repeated cycles of protein solution application and drying. We directly compare the protein level, capture DNA level, and target DNA binding signal for the two most promising anchor proteins. Finally, for our chosen loading level of protein anchor, we demonstrate a sandwich format assay for ssDNA (representing, for example, single-stranded amplicons from an asymmetric PCR amplification) in the context of a SARS-CoV-2 sequence, and achieve a limit of detection of 0.5 nM and 0.2 nM using gold nanospheres and gold nanoshells, respectively, as labels.

## 2. Materials and Methods

### 2.1. Chemicals and Biochemicals

The four biotin-binding proteins investigated were Polystreptavidin R (#10120030, Biotez, Berlin, Germany), nitrocellulose-binding streptavidin (PRO-338, Prospec, Revohot, Israel), anti-biotin antibody (monoclonal, Z021, Invitrogen, Carlsbad, CA, USA), and streptavidin (189730, EMD Millipore). The DNA sequences with or without biotin or FAM were purchased from a commercial vendor (IDT, Integrated DNA Technologies, Inc., Coralville, IA, USA) as summarized in Table 1. The sequence of our synthetic DNA is from the SARS-CoV-2 virus. The rinse and running buffers used were composed of standard ingredients in a polymerase chain reaction buffer, including 10 mM Tris, 50 mM KCl, and 1.5 mM MgCl_2_ but without the polymerase enzymes, primers, or free nucleotides.

### 2.2. Materials and General Device Fabrication

The materials consisted of nitrocellulose (FF120HP, Whatman, Cytiva, Marlborough, MA, USA), cellulose (CFSP22300, EMD Millipore, St. Louis, MO, USA), glass fiber (Ahlstrom, 8951 chopped glass with binder), and polymeric laminates with an adhesive (Tekra Corporation, New Berlin, WI, USA). Manufacturer-reported specifications for FF120HP include a thickness of 100 μm (on a 100 μm polyester backing) and a capillary rise time of 90 to 150 s per 4 cm. Further, the porosity of the membrane was measured to be ~70% [19]. A CO_2_ laser system (Universal, VLS 3.5, Scottsdale, AZ, USA) was used to cut the materials to size. Capture reagents were applied onto the nitrocellulose either via hand-spotting using a small-volume pipette or striping using a liquid dispensing system (AD3320, BioDot, Irvine, CA, USA). Nitrocellulose strips were placed on 10 mil thick polyester with an adhesive base and then the cellulose pad was placed downstream and overlapped the nitrocellulose by 2 mm. A layer of 10 mil thick polyester was placed over the cellulose pads to facilitate contact between the cellulose pads and the nitrocellulose strips, and spacers were utilized to support uniform contact across the multiple strips in a device. Glass fiber pads were attached to the upstream end of the nitrocellulose strips for any devices involving target nucleic acid hybridization.

### 2.3. Evaluating Adsorption of Protein Anchor on Nitrocellulose Using Hand-Spotting (Figure 2Ai,Aii)

Protein solutions of pSA and NC-SA were created at a concentration of 1 mg/mL using 10 mM phosphate-buffered saline (PBS) (Sigma Aldrich, St. Louis, MO, USA) at pH 7.4 as the diluent. The anti-biotin antibody, in an amount of 1 mg/mL in 10 mM PBS with 0.1% sodium azide, was used directly, and streptavidin was resuspended in an amount of 1 mg/mL in PBS. Each protein solution was hand-spotted (0.5 μL) onto a nitrocellulose strip that was 0.3 cm wide at a distance of 1.8 cm from the upstream edge of the strip, and then allowed to dry overnight in a desiccator. For the rinse conditions of the buffer or buffer with the nonionic surfactant Tween 20 at 0.1%, 100 μL of the rinse fluid was added to the wells of a 96 well plate and the nitrocellulose strips, attached to a support, were placed in the wells for 5 min. The strips were allowed to dry for 30 min and then protein-stained as described below. For comparison, the case of no rinse was also assessed. Images were analyzed to obtain the average background-corrected greyscale intensity of the protein stain for each strip for the different cases. Replicates of four strips were evaluated for each case and the average and standard deviation were calculated.

### 2.4. Evaluating Adsorption of Capture Reagents on Nitrocellulose after Hand-Spotting (Figure 2Bi–Biii)

Protein solutions at a final concentration of 0.2 mg/mL were mixed with biotinylated capture DNA at concentrations estimated to be approximately double the number of available biotin-binding sites in the protein sample and allowed to incubate for 30 min. The biotinylated capture DNA concentrations were 10 μM, 30 μM, 20 μM, and 30 μM, for anti-biotin IgG, SA, NC-SA, and pSA, respectively. Each solution was hand-spotted (0.5 μL) at a location 1.8 cm from the downstream edge of a nitrocellulose strip that was 0.3 cm wide, and then allowed to dry overnight in a desiccator. Each membrane, attached to a support, was rinsed by placing the nitrocellulose strip into a well of a 96-well plate filled with 100 μL of the buffer plus 0.1% Tween 20, and allowing the rinse fluid to flow for 5 min. Four replicates of each protein anchor on nitrocellulose were evaluated using protein stain and DNA stain as described below, for the cases of no rinse and after a rinse.

We evaluated the deposition of multiple passes of capture reagents on nitrocellulose using the BioDot liquid dispensing system (Figure 3). Capture reagents were premixed at a concentration of 2 mg/mL pSA and 250 µM biotinylated capture DNA in PBS and incubated for 1 h. The BioDot liquid dispensing system was used to apply one pass, two passes, three passes, and four passes of the capture reagents striped onto nitrocellulose (with 10 min of drying time between each pass). The substrate was dried overnight in a desiccator and cut into strips. The strips were rinsed to remove excess capture NA and any unbound pSA via two applications of 15 μL of PCR buffer with 0.1% Tween 20 and then dried for 1 h. Three replicate striped membranes were evaluated for protein and DNA using staining as described below.

An additional set of striped membranes were investigated for target DNA binding at 50 °C. Solutions (15 μL) of fluorescently labeled target DNA in PCR buffer, at 0.5 μM and 0 μM, were applied to glass fiber sample pads at the upstream edge of the nitrocellulose and allowed to flow for 2 min. A final rinse with 30 μL of PCR buffer with 0.1% Tween 20 was applied to the glass fiber pads (in two applications of 15 μL), and then the strips were dried for 1 h in a covered desiccator. The strips were imaged under a blue light illuminator and analyzed as described below. These images were analyzed using a custom MATLAB program as further described below.

We evaluated target NA binding signal to capture NA using striped pSA and NC-SA protein anchor systems on nitrocellulose (Figure 4). Capture reagent solutions were premixed at a concentration of 1.5 mg/mL of pSA with 180 µM biotinylated capture DNA or 1.5 mg/mL of NC-SA with either 126 µM or 252 µM biotinylated capture DNA, and incubated for 1 h. The BioDot liquid dispensing system was used to apply three passes of a solution to nitrocellulose membranes. The membranes were then dried overnight in a desiccator and cut into strips. The nitrocellulose strips were attached to a polyester and adhesive support, and glass fiber pads were added to the upstream end of the nitrocellulose strips with a 0.3 cm overlap. The strips were rinsed to remove excess capture NA and any unbound pSA via the application of 30 μL of PCR buffer with 0.1% Tween 20 to each of the glass fiber pads. The strips were dried for 1 h in a desiccator. Solutions of fluorescently labeled target DNA in PCR buffer were created at concentrations of 0.5 μM and 0.25 μM via serial dilution (0 μM was also considered). For both proteins, three replicates of each fluorescently labeled target DNA concentration were hybridized at 50 °C by applying 15 μL of the fluorescently labeled target DNA solution to the glass fiber pads and allowing it to flow for 2 min. A final rinse with 30 μL of PCR buffer with 0.1% Tween 20 was applied to the glass fiber pads and allowed to flow for 10 min, before the strips were dried for 1 h in a covered desiccator. The strips were imaged under a blue light illuminator and the images were analyzed using a custom MATLAB program as described below.

### 2.5. Sandwich Assay with Gold Nanoparticle Labels (Figure 5 and Figure 6)

The capture reagents, 2.0 mg/mL pSA and 250 μΜ biotinylated capture DNA in PBS, were incubated for 1 h. The BioDot liquid dispensing system was used to apply 3 passes of the capture reagents on the nitrocellulose substrates; the substrates were dried overnight in a desiccator, and then cut into strips (1.6 cm long) using a CO_2_ laser. The strip varied in width from a maximum of 3 mm at the upstream and downstream ends and a minimum of 1 mm in the detection region. Gold nanoparticles, either 40 nm diameter nanospheres functionalized with streptavidin (nanoComposix, San Diego, CA, USA) at OD 5, or 150 nm gold nanoshells functionalized with streptavidin (nanoComposix, San Diego, CA, USA) at OD 5, were incubated with 6.7 μM biotinylated label DNA or 0.53 μM biotinylated label DNA, respectively, for 1 h and then centrifuged. The pelleted material was recovered to create a gold conjugate-label DNA solution that also contained 0.5% Tween 20, 5% sucrose, and 2.5% BSA. A volume of 10 μL of the suspension was applied to 10 μL capacity glass fiber pads and the pads were dried under a vacuum for 1 h at 37 °C. The nitrocellulose strips were attached to a polyester and adhesive support, and the glass fiber pads were added to the upstream end of the nitrocellulose strips with a 0.3 cm overlap (for the nanoshell assay, a post-deposition rinse of 20 μL of PBS with Tween 20 was applied to remove excess capture reagents). Solutions of target DNA, at 16 nM, 8 nM, 4 nM, 2 nM, 1 nM, 0.5 nM, and 0 nM for the assay with nanospheres, and 4 nM, 2 nM, 1 nM, 0.5 nM, 0.25 nM, 0.125 nM, and 0 nM for the assay with nanoshells, in PCR buffer with Tween 20, were created via serial dilution. A concentration of 0.25% Tween 20 was used in the nanosphere assay and a higher concentration of 0.5% Tween 20 was used in the nanoshell assay. Each target DNA solution and the control solution were assayed at 50 °C by applying 10 μL to the glass fiber pad containing the dried conjugate and allowing it to flow for 3.5 min. Two rinses of 10 μL of PCR buffer with Tween 20 (either 0.25% or 0.5%) were then applied to the glass fiber pads and allowed to flow for 3.5 min each. The cards were imaged while the strips were still wet using a scanner (Epson V700) to obtain 300 ppi, three-channel, 48-bit TIF images. The images were analyzed using a custom MATLAB program as described below.

### 2.6. Striping of Capture Reagents on Nitrocellulose

The BioDot liquid dispensing system was used to dispense capture reagents onto nitrocellulose. Briefly, a line dispense script was developed in the BioDot AxSys script program to stripe multiple passes of solutions onto nitrocellulose by using a loop that would aspirate and stripe one pass of the solution, purging excess solution, and then drying it for 10 min before repeating a specified number of times. The solution was dispensed at 0.50 μL/cm allowing for 0.15 μL or 0.05 μL to be applied to each strip per pass for 3 mm wide or 1 mm wide strips, respectively. An open time of 530 μs, two pre-dispense drop loops of 20 × 40 nL each, and a drop volume of 0.02 μL were used. Once striping was completed, the substrates were allowed to dry overnight in a desiccator. All substrate material was cut to the desired shape and size using the CO_2_ laser system. Multi-strip cards were fabricated from nitrocellulose and polyester with adhesive layers.

### 2.7. Protein Visualization

Proteins were visualized on nitrocellulose using the Pierce Blue protein stain (#24580, Thermo Fisher Scientific, Waltham, MA, USA). The membranes were rinsed with deionized (DI) water, rinsed with the stain for ~5 min, rinsed with destaining for ~5 min, and rinsed again with DI water. The membranes were dried for approximately one hour before being imaged with a scanner (Epson V700) to obtain 300 ppi, three-channel, 24-bit or 48-bit TIF images.

### 2.8. DNA Visualization

DNA on nitrocellulose was visualized, either with the methylene blue stain or the fluorescence from the carboxyfluorescein (FAM) that had been attached to the DNA sequence of interest. For methylene blue staining, the membranes were gently rocked while submerged in methylene blue for 5 min. The stain was decanted, the membranes were washed with DI water for 20 s, and the latter was repeated with fresh DI water for a total of 3 washes. The membranes were dried for 1 h before being imaged with a scanner (Epson V700) to obtain 300 ppi, three-channel, 24-bit or 48-bit TIF images.

### 2.9. FAM Visualization

For FAM visualization, the membranes were placed in a blue light illuminator (MAESTROGEN, Taiwan) and RAW images were taken with a Samsung smartphone camera (Galaxy S9+) using the app OpenCamera (https://opencamera.org.uk/, 2022) with ISO settings of 250 and a shutter speed of 1/40 s.

### 2.10. Image Analysis

A custom MATLAB (MathWorks, Natick, MA, USA) script was used to quantify the average greyscale intensity for protein or DNA staining in a region of interest (ROI). First, the ROI was manually selected in the image. For the hand-spotted membranes, the ROIs were circular (diameter of 0.16 cm), and the center of the capture region was chosen for analysis. For the striped membranes, the ROI was rectangular (0.23 cm × 0.04 cm for the full-width strips or 0.04 cm × 0.04 cm for the narrow ones), and the center of the rectangle was selected. Within each ROI, the RGB values for each pixel were converted into greyscale via averaging. This was also carried out for a same-sized regions 0.5 cm (for hand-spotted membranes) or 0.15 cm (for striped membranes) upstream, in order to obtain an estimate of the background intensity. For the hand-spotted protein and DNA stain data, the background-corrected intensity was calculated as the average intensity in the detection ROI subtracted from the average intensity in the background ROI, and then reported as the signal. For the striped protein and DNA stain data, the normalized signal was calculated as the background-corrected intensity divided by the average intensity in the background ROI. For the fluorescence data, the normalized signal was calculated as the background-corrected intensity, the average intensity in the background ROI subtracted from the average intensity in the detection ROI, and then divided by the quantity, the average intensity in the background ROI subtracted from 2^16^. The normalized signal for the lateral flow assay data was calculated in the same manner as carried out for the striped protein and DNA stain data. 

Variability in the hand-spotting procedure was estimated by measuring the areas of the stained regions using Image J (1.53k, http://imagej.nih.gov/ij) and calculating the coefficient of variation in area. 

### 2.11. Limit of Detection Estimation

The limit of detection (LOD) of the assay was calculated using the following equation: $signal\;LOD=\mu_{zero}+1.645\sigma_{zero}+1.645\sigma_{low}$, where $\;\mu_{zero}$ is the average signal of the no-target DNA control, $\sigma_{zero}$ is the standard deviation of the no-target DNA control, and $\sigma_{low}$ is the standard deviation of the lowest non-zero target DNA concentration signal. The concentration LOD was then estimated assuming a linear calibration curve, $concentration\;LOD=\frac{[{µ}_{blank}+1.645\left(\sigma_{blank}+\sigma_{low}\right)]-b}{m}$, where m is the slope and b is the intercept (obtained from a linear regression of the experimental data for data points from 0 to 2 nM target DNA).

## 3. Results and Discussion

### 3.1. Protein Characteristics to Consider in Creating a High Density of Biotin-Binding Sites on Nitrocellulose

Key characteristics of the four biotin-binding protein anchors, schematically shown in Figure 1, are listed in Table 2. One of the characteristics critical in targeting nucleic acid binding is the number of biotin-binding sites per mass unit of protein (estimated using the number of potential biotin-binding sites per molecular unit and the molecular weight of the protein). When considering the protein in solution (i.e., no orientation dependence), streptavidin with four binding sites per 60 kD molecular units has the greatest number of biotin-binding sites per mass unit. Polystreptavidin R (pSA), advertised as a ‘chemically modified polymerized streptavidin’, is assumed to have the next-highest number of binding sites per mass unit. This is followed by nitrocellulose-binding streptavidin (NC-SA), which exists as a monomer with a MW of 24 kD. Lastly, IgG antibody (Ab) has an approximate MW of 150 kD. Note that for the heterogenous lateral flow assays of interest here, the orientation of the adsorbed protein and specifically, the accessibility of the biotin-binding sites in the adsorbed protein will be substantially reduced with the possible exception of NC-SA. For IgG antibody, it is known that nonspecifically adsorbed IgG has reduced binding in variable regions [12] and efforts have been devoted to creating a nitrocellulose-binding version of protein A (that binds the constant region of IgG) to improve binding site accessibility [20]. Less has been reported about SA orientation after adsorption to nitrocellulose, but given the symmetric nature of SA, adsorption in any orientation would be expected to substantially reduce the accessibility of biotin-binding sites. A similar reduction would be expected for pSA. For NC-SA, the design of the recombinant protein is likely such that the nitrocellulose-binding domain is located away from the biotin-binding domain.

Another key consideration is the surface density with which the protein anchor attaches to nitrocellulose, which in turn depends on the protein adsorption affinity to nitrocellulose (or the complex of protein attached to capture nucleic acid, if premixing) and overall protein loading onto the substrate. For example, although not often reported as practiced, additional protein can be loaded onto the substrate through multiple applications of the capture reagent and may partially compensate for lower-affinity adsorption. Of course, additional loading requires additional time, comes with the possibility of nonuniform loading, and involves an added cost of reagents. Thus, investigations of these issues in the context of nucleic acid lateral flow detection can inform assay development as described below.

### 3.2. Robustness of Protein Attachment to Nitrocellulose

**Figure 2:**
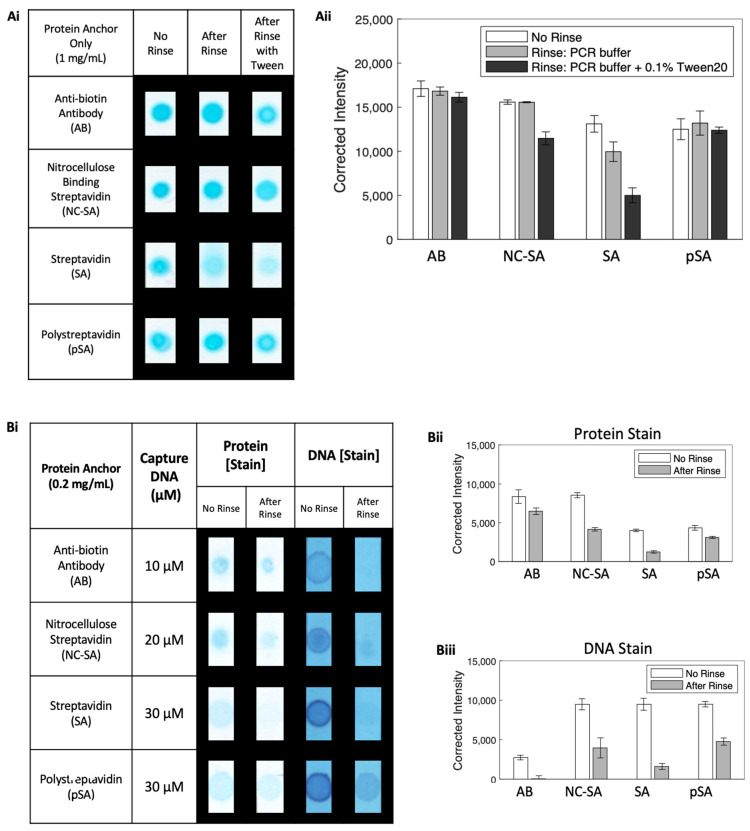
DNA capture using protein anchor immobilization on nitrocellulose with direct adsorption. (**Ai,Aii**) Proteins (1 mg/mL) on nitrocellulose exposed to one of three conditions, no rinse, buffer rinse, or buffer plus Tween 20 rinse, and then subsequently assessed for protein. (**Ai**) Images of stained protein for each condition. (**Aii**) Bar chart of the average background-corrected intensity (*N* = 3) for the different anchor proteins and different conditions. (**Bi–Biii**) Proteins (0.2 mg/mL) premixed with biotinylated DNA (various concentrations) and then applied to nitrocellulose exposed to one of two conditions, no rinse or buffer plus Tween 20 rinse, and then subsequently assessed for protein and capture DNA. (**Bi**) Representative images of stained protein, stained DNA, and fluorescent target DNA for each condition. (**Bii**) Bar chart of the average background-corrected intensity from protein staining (*N* = 4) for each protein anchor and condition. (**Biii**) Bar chart of the average background-corrected intensity from the DNA staining of capture DNA (*N* = 4) for each protein anchor and condition. Bars are the average of replicates and error bars represent the standard deviation.

We investigated the immobilization to nitrocellulose of four different anchor proteins (0.5 µg mass) that could be used to bind biotinylated nucleic acid capture sequences, with the results shown in Figure 2Ai,Aii. These constituted (i) anti-biotin immunoglobulin (IgG), (ii) streptavidin (SA), (iii) nitrocellulose-binding streptavidin (NC-SA), and (iv) Polystreptavidin R (pSA). Images of the replicate protein-stained membranes for each protein are shown in Figure 2Ai and a plot of the background-corrected intensity for the different protein anchors and rinse cases is shown in Figure 2Aii. IgG is well known to adsorb robustly to nitrocellulose [21], and as expected, the anti-biotin IgG produced a high-intensity protein stain signal for all conditions of no rinse, after rinse with buffer, and after rinse with buffer with Tween 20. The SA protein showed a strong protein stain signal on nitrocellulose for no flow, but then a significantly lower signal after buffer flow and an even lower signal after buffer with Tween 20 flow. The NC-SA showed strong signals for no rinse and after buffer rinse, and a somewhat reduced signal after buffer rinse with Tween 20. These results are in agreement with reports from Holstein [12,13]. The fourth anchor, pSA, that previously has not been compared directly with these others, showed robust adsorption in all cases of no flow, after buffer rinse, and after buffer rinse with Tween 20. Specifically, pSA is similarly robust under buffer rinse with Tween 20 to IgG. As a measure of hand-spotting variability, we found an average coefficient of variation of 16% (with a range of 11% to 20%) for the hand-spotted protein areas with no rinse. Further, the % difference in spotted protein areas from the largest case of Ab was 8% for NC-SA, 9% for pSA, and 16% for SA. Although the variability in hand-spotting could be a limitation for the measurement of small quantitative differences in stain signals across proteins, it does enable us to highlight the substantial differences in the robustness of protein adsorption onto nitrocellulose before vs. after rinsing with and without the surfactant (as demonstrated in the bar chart of Figure 2Aii).

### 3.3. Robustness of Attachment of Protein–DNA Complex to Nitrocellulose after Premixing

We then investigated the level of immobilization to nitrocellulose of each of the four different anchor proteins that had been premixed with biotinylated nucleic acid capture sequences, with the results shown in Figure 2Bi–Biii. Since our capture reagent population that was applied to the nitrocellulose was composed of a protein anchor and biotinylated capture DNA that was bound in a complex as well as free, both the level of protein and the level of nucleic acid was assessed using different stains (as described in the Materials and Methods). Images of the replicate protein- and DNA-stained membranes for the different cases are shown in Figure 2Bi, while the plots of background-corrected intensity for protein and capture DNA are shown in Figure 2Bii and Figure 2Biii, respectively. Note that the concentrations of the biotin-binding protein anchor and the biotinylated capture DNA are at lower concentrations than would likely be used to create capture surfaces for a high-sensitivity test. The lower concentrations were chosen to be well below the saturation ranges of both the protein stain and DNA stain to ensure an accurate quantification of differences in protein and DNA levels among the different anchor systems. Further, for each protein anchor, the number of biotinylated capture DNA sequences added to the premix containing 0.1 µg of protein was chosen to be equal to twice the number of biotin-binding sites available in that mass of protein (see Figure S1 in Supplementary Information for pSA). The rationale for this ratio (of 2:1 of the biotinylated DNA sequence to the number of biotin-binding sites in the protein) was to ensure an excess of biotinylated DNA to available biotin-binding sites on the protein that would drive binding between the two, while not having such an excess of free biotinylated DNA that it would interfere with protein association with and adsorption to the nitrocellulose.

An assessment of the protein levels for the different protein anchors after premixing with biotinylated DNA indicated that in some cases, the addition/presence of DNA affected the level of protein that was adsorbed onto the nitrocellulose. Specifically, both the SA and pSA that were premixed with biotinylated DNA appeared to have a lower affinity to nitrocellulose (leftmost two columns of images in Figure 2Bi) compared to those for proteins only (leftmost two columns of images in Figure 2Ai) as indicated by the greater spread in the signal when the protein was premixed vs. when only the protein was deposited. Note that although the concentration of protein was higher in the protein-only case than in the premix-protein-plus-biotinylated-DNA case, the protein-plus-biotinylated-DNA mixture resulted in a larger diameter protein spot than that in the protein-only case for SA and for pSA. This indicates that the presence of biotinylated DNA with the protein interfered with the adsorption of proteins SA and pSA to the nitrocellulose. In contrast, the protein signal for NC-SA, after being premixed with biotinylated DNA, did not substantially change in lateral area (leftmost two columns of images in Figure 2Bi) relative to the signal for the protein-only case (leftmost two columns of images in Figure 2Ai).

An assessment of capture DNA levels for the different protein anchors indicated that the protein anchors provided different levels of capture DNA immobilization as seen in the DNA stain before and after rinsing. The DNA stain signal before rinsing included both biotinylated DNA bound to protein and unbound biotinylated DNA. As DNA has a low affinity to nitrocellulose, the radial extent of the signal matched that of the front of the fluid volume deposited onto the nitrocellulose substrate. The DNA stain signal after rinsing included the biotinylated DNA that was bound to the protein only (or any nonspecifically adsorbed biotinylated DNA) and was similar in lateral area to that of the protein signal for each NC-SA, SA, and pSA. NC-SA and pSA displayed the highest levels of capture DNA immobilization. In contrast, the SA anchor produced only a low-level DNA signal (after rinsing), while anti-biotin IgG produced no DNA signal. The low level of DNA immobilization for SA was expected, given the poor level of SA immobilization to nitrocellulose. However, the anti-biotin IgG result was not expected, and indicated that the particular antibody chosen had a poor affinity to biotin. It is possible the DNA signal for IgG could be increased by using a higher biotin–DNA concentration and a longer incubation time, but this would add to the cost of the method. Based on these results from hand-spotting protein anchor systems onto nitrocellulose, the most promising proteins that provided the highest levels of protein and capture DNA on nitrocellulose were pSA and NC-SA. Although hand-spotting can be an effective tool for screening and preliminary work, the use of automated reagent dispensing systems can be critical for improved reproducibility in assay development. Further, these automated systems can enable multiple passes of reagent application onto the substrate to achieve higher-density capture surfaces that can be critical for high-sensitivity assays. This is especially useful in cases where the protein concentration is limited by the vendor, i.e., many antibodies are sold at a concentration of 1 mg/mL. Although hand-spotting can be a time- and resource-efficient way to apply reagents to substrates, the method is prone to higher variability compared to that for automated dispensing systems. Thus, for the next stage in our study, we transitioned to investigating protein anchor application using an automated dispensing system, first focusing on pSA with multiple passes of protein anchor application followed by a direct comparison of pSA with NC-SA anchors on nitrocellulose.

### 3.4. Evaluating Protein Anchor Immobilization on Nitrocellulose Using Multiple Passes of Reagent Deposition

**Figure 3:**
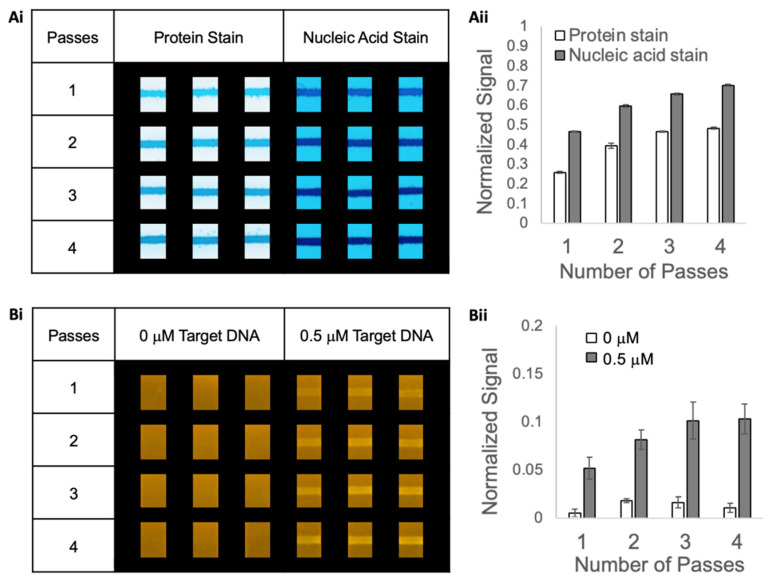
DNA attachment using protein (pSA) anchor immobilization on nitrocellulose with multiple passes of capture reagent application. Polystreptavidin R (2 mg/mL) premixed with biotinylated capture nucleic acid (250 µM) was striped onto nitrocellulose with 1 pass, 2 passes, 3 passes, and 4 passes, rinsed with buffer plus Tween 20, and then assessed for the presence of protein and nucleic acid. (**Ai**) Images of the protein- and nucleic acid-stained strips. The dimensions of the cropped images are 2.4 mm wide and 3.2 mm tall. (**Aii**) Bar chart of the average normalized signal (*N* = 3). Error bars represent the standard deviation. (**Bi**) Images of the FAM-labeled DNA in the detection region of the strips. The dimensions of the cropped images are 2.4 mm wide and 3.2 mm tall. (**Bii**) Bar chart of the average normalized signal (*N* = 3). Error bars represent the standard deviation.

We assessed the effect of striping multiple passes of pSA premixed with biotinylated DNA onto nitrocellulose and the results are shown in Figure 3Ai,Aii. Images of the protein- and DNA-stained substrates are shown in Figure 3Ai and plots of normalized signals for protein and DNA are shown in Figure 3Aii. Note that a key requirement for creating high-density capture surfaces using multiple passes of reagent deposition is to allow an adequate drying time between each pass such that additional protein-DNA has the chance to adsorb within the original region of interest rather than being transported to regions of nitrocellulose outside of the original region of interest (a consequence if the substrate is not dry) and substantially increasing the area of protein immobilization. We estimate that each pass deposited approximately 0.3 µg of protein (0.05 µL/mm at a dispense rate of 2.0 mg/mL protein across 3 mm). Not all the deposited protein was expected to adsorb onto the nitrocellulose, so we expect that the post-deposition substrate contained adsorbed protein either bound to capture DNA or not, as well as non-adsorbed protein (also bound to capture DNA or not) and free biotinylated capture DNA. Thus, a post-deposition rinse, using lateral flow through the membrane, was implemented to remove the non-adsorbed protein and free biotinylated capture DNA from the substrate.

The average signal of protein on the nitrocellulose membrane increased after each additional pass from one to four passes, as shown in Figure 3Ai. However, the gain in signal decreased with an increasing number of passes and the signal was comparable for three and four passes. Note, however, that we did not expect the protein stain signal response to be linear with the concentration across this range, and that a nonlinear response would decrease in sensitivity at higher concentrations. Similarly, the average signal of capture DNA on the nitrocellulose increased with an increasing number of passes, as shown in Figure 3Aii. The gain in signal for the capture DNA also decreased with an increasing number of passes with only a minor increase between three and four passes. As expected, this is consistent with the trends observed in protein signals. Since each additional pass of capture reagent deposition requires time for patterning and for drying, as well as the additional cost of reagents, a direct evaluation of target DNA binding is also critical to evaluate. Hence, next, we evaluated the level of target DNA binding to each of the different capture surfaces with the results shown in Figure 3Bi,Bii. Images of the substrates after the application of 0.5 µM target DNA (FAM-labeled) or of negative controls with no target applied are shown in Figure 3Bi and plots of the normalized signal for the fluorescent target DNA are shown in Figure 3Bii. Since no FAM-labeled target DNA was exposed to the substrates for the negative control samples, we speculate that the low-level zero signals were caused by minor variations in background fluorescence across the substrates. The average signal of target DNA captured significantly increased with an increasing number of passes between one and three passes, but then leveled off, which is consistent with the trends observed for both the protein anchor and capture DNA. Taken together, the data indicate that the level of protein anchor and capture DNA adsorbed onto the nitrocellulose was comparable for three and four passes and likely near saturation under these conditions. Thus, the condition of three passes of capture reagent deposition was chosen for our further work (note that depending on the application requirements, fewer passes and lower capture DNA concentrations might satisfy assay sensitivity requirements with time and reagent cost savings).

### 3.5. Comparison of pSA and NC-SA as Protein Anchors Using Automated Liquid Dispensing for Protein Anchor Application

**Figure 4:**
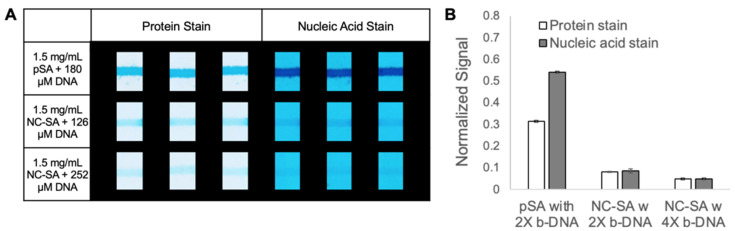
Direct comparison of pSA and NC-SA anchor systems applied onto nitrocellulose substrate using a liquid dispensing system. Three passes of capture reagent solutions composed of pSA (1.5 mg/mL) and biotinylated capture DNA with twice the estimated binding sites (180 μM), NC-SA and capture DNA with twice the estimated binding sites (126 μM), and capture DNA at four times the estimated binding sites (252 μM) were applied and assessed for the presence of protein and nucleic acid with the Pierce Blue protein stain and Methylene Blue nucleic acid stain. (**A**) Protein and nucleic acid staining results. The cropped viewing region is 2.4 mm wide and 3.2 mm tall. (**B**) Bar chart of the average normalized signal (*N* = 3). Error bars represent the standard deviation.

Next, we directly compared the performance of pSA and NC-SA as protein anchors premixed with biotinylated nucleic acid for (i) protein levels and (ii) capture NA levels with the results shown in Figure 4. Specifically, the same mass of either pSA or NC-SA premixed with biotinylated capture DNA at approximately two times the estimated number of biotin-binding sites was applied using an automated liquid dispensing system with our chosen three passes of capture reagents. Additionally, for NC-SA, premixing with approximately four times the estimated number of biotin-binding sites was investigated. Both pSA and NC-SA displayed appreciable protein stain signals, but the pSA protein signal was also substantially higher than that of NC-SA as shown in Figure 4A. Since the same mass of protein was deposited in each case, the data indicate that NC-SA protein when premixed with an excess of biotinylated DNA is less effective at adsorbing onto nitrocellulose than is pSA and that adsorption efficiency decreases with an increasing amount of premixed biotinylated capture DNA. The data suggest that the presence of excess capture DNA interferes with NC-SA adsorption onto nitrocellulose. NC-SA bound to capture DNA may also have reduced the efficiency of adsorption onto nitrocellulose compared to the free protein, but this was not directly assessed. The nucleic acid stain data shown in Figure 4B mirror the protein stain data, with the pSA anchor system outperforming NC-SA, and a substantially lower level of capture DNA being left on the substrate after premixing with a higher level of excess biotinylated capture DNA. An additional reason for the lower capture NA signal for NC-SA is that NC-SA is estimated to have a lower number of biotin-binding sites than pSA has for the same mass of protein. Regarding target DNA capture from these capture surfaces, the pSA anchor resulted in a strong target DNA fluorescence signal from the FAM label, while the NC-SA anchor resulted in no detectable fluorescence signal (see Figure S2 in Supplementary Information). Although NC-SA did not perform well as a protein anchor using a one-step protocol for capture reagent deposition, NC-SA may be well-suited to a two-step process in which NC-SA is first allowed to adsorb (i.e., NC-SA showed strong adsorption to nitrocellulose in Figure 2Ai,Aii) and then biotinylated capture DNA is deposited for attachment to free biotin sites. Given the high-density capture reagent loading demonstrated with pSA, the much higher cost of NC-SA relative to that of pSA, and the potential requirement for additional processing steps for NC-SA (however, note that an alternative would be to have the target DNA bind both the capture DNA as well as the label DNA upstream of the detection region), NC-SA was not investigated further.

### 3.6. Demonstrating Multi-Layer pSA-Capture DNA Surfaces for Nucleic Acid Detection in a Lateral Flow Assay

**Figure 5:**
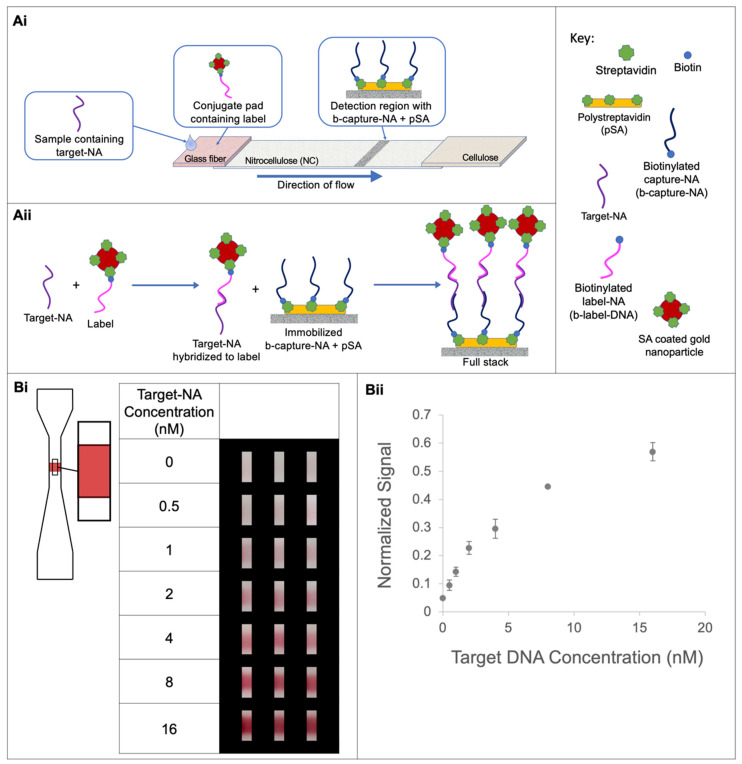
Assay response with dried down gold nanospheres. (**Ai**) Diagram of lateral flow assay format. (**Aii**) Diagram of full stack hybridization scheme. (**Bi**) Image data for each target-DNA concentration cropped to remove edge effects. The strip is 1.6 cm long, the unnarrowed region of the strip is 3 mm wide, the narrowed region of the strip is 1 mm wide, and the cropped region is ~0.4 mm wide and ~1.3 mm tall. (**Bii**) Assay response for target DNA concentrations from 0 to 16 nM. Data points are the average of replicates (*N* = 3) and error bars represent the standard deviation.

**Figure 6:**
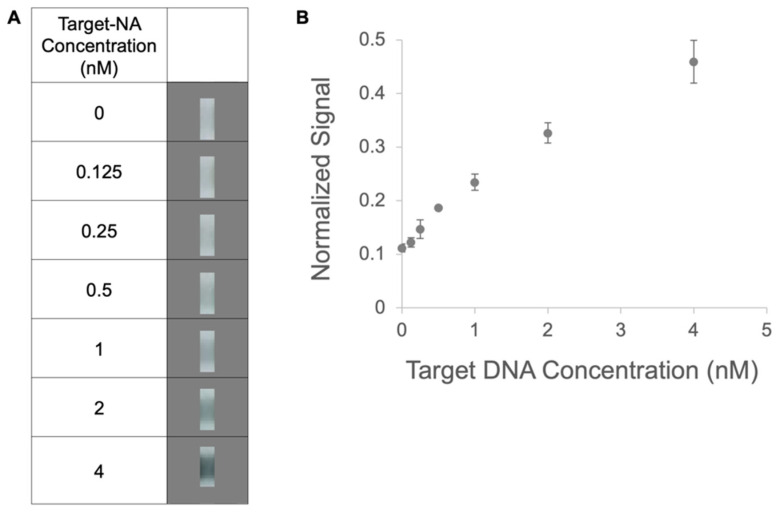
Assay response in full stack with dried gold nanoshells. (**A**) Representative image data for each target-DNA concentration cropped to remove edge effects. The strip is 1.6 cm long, the unnarrowed region of the strip is 3 mm wide, the narrowed region of the strip is 1 mm wide, and the cropped region is ~0.5 mm wide and ~1.5 mm tall. (**B**) Assay response for target DNA concentrations from 0 to 4 nM. Data points are the average of replicates (*N* = 3 or 2) and error bars represent the standard deviation.

Next, we assessed the performance of pSA as a protein anchor in a lateral flow, sandwich-format assay, as schematically shown in Figure 5Ai,Aii. The response of the assay and the assay’s limit of detection (LOD) were characterized. The assay design employed a nitrocellulose strip with a narrowed detection region, a sample input volume of 10 μL, and a total run time of 10.5 min from sample addition to imaging. Note that the assay was performed at an elevated temperature of 50 °C, in anticipation of future work with real-world samples and for which higher temperature hybridization would suppress nonspecific nucleic acid binding events. Further, this temperature was chosen to be below the predicted melting temperature of our target–capture nucleic acid complexes and below the temperatures at which an appreciable dissociation of biotin and streptavidin would be expected to occur in a salt-containing buffer [22]. Based on the above results (Figure 3Ai,Aii), three passes of premixed capture reagents (2.0 mg/mL of pSA with biotinylated capture DNA at over double the estimated number of biotin-binding sites, 250 μM) was applied using the liquid dispensing system in order to obtain a robust, high-density capture surface. The motivation for narrowing the detection region of the strip was to expose the sample to a smaller detection volume and achieve potentially improved binding to the capture DNA relative to a constant width strip (demonstrated in [23]). The target sample and two subsequent rinses (10 μL each) were applied sequentially with 3.5 min between applications, a period found to enable the emptying of the majority of the fluid in the conjugate pads. The image data in Figure 5Bi show low nonspecific adsorption for the negative control with no target DNA and increasing signals with increasing concentrations of target DNA for the positive samples. The assay response, shown in the plot of Figure 5Bii, indicates a linear range below 2 nM. The assay concentration’s LOD using the gold nanosphere labels was estimated to be ~0.5 nM (3 × 10^9^ copies in 10 μL). We further characterized a version of our assay using gold nanoshells, which have the advantage of appearing as high-contrast black on a white nitrocellulose background. Image data are shown in Figure 6A and a plot of the response curve is shown in Figure 6B. The assay is linear over the range investigated from 0 to 4 nM with a concentration LOD of 0.2 nM (1.2 × 10^9^ copies or 2 fmol in 10 μL), an improvement of over two times that achieved using nanosphere labels. It is not possible to directly compare these results to other work due to the many unmatched conditions between studies, but a survey of the literature indicates that these results compare reasonably with those reported for DNA detection using gold labels in a lateral flow format. For example, Glynou et al. reported 0.4 nM (2 fmol target in 5 μL) DNA detection using their lateral flow assay with gold labels [11], while Rastogi et al. reported the detection of ~3 nM DNA with unmodified gold nanoparticles in their sandwich-format assay, and an improved LOD of ≤0.4 nM DNA with their modified nanoparticle system (using locked nucleic acids) [24]. Note that we chose to limit the sample volume and processing time to be compatible with field-use assay parameters, but additional improvements in LOD could be achieved at the expense of an additional sample volume and processing time.

## 4. Summary

In this work, we have investigated multiple biotin-binding proteins as anchors for use in lateral-flow-format assays for nucleic acid detection. We have systematically benchmarked capture reagent adsorption/binding to a nitrocellulose substrate and subsequent target DNA capture for different levels of capture reagent loading. Further, we have used our characterization to choose the best balance of signal vs. the additional time and cost of capture reagents. Finally, we have demonstrated our high-density capture surfaces in high-sensitivity COVID-19 DNA lateral flow assays using two different types of gold nanoparticles, nanospheres and nanoshells, with a limit of detection of 0.5 nM and 0.2 nM, respectively.

## Figures and Tables

**Table 1:** DNA sequences used in this study (modified from [18]).

Name	DNA Sequence (5’–3’)
Target DNA with FAM	FAM-ACACTAGCCATCCTTACTGCGCTTCGATTGTGTGCGTACTGCTGCAATATTG
Target DNA	ACACTAGCCATCCTTACTGCGCTTCGATTGTGTGCGTACTGCTGCAATATTG
Biotin-labeled capture DNA (with 35 A spacer)	Biotin-AAAAAAAAAAAAAAAAAAAAAAAAAAAAAAAAAAAATATTGCAGCAGTACGCACACA
Biotin-labeled label DNA (with 35 A spacer)	GCGCAGTAAGGATGGCTAGTGTAAAAAAAAAAAAAAAAAAAAAAAAAAAAAAAAAAA-Biotin

**Table 2:** Characteristics of the biotin-binding species used in protein-mediated DNA sequence attachment to nitrocellulose.

Protein anchor	Molecular Weight (kD)	Biotin-Binding Sites per Molecular Unit and Relative to SA	Estimated Biotin-Binding Sites per 0.5 μg of Protein Mass	Estimated Biotin-Binding Sites per 0.5 Μg Of Protein Adsorbed Mass	Packing Density Relative to SA	Cost per 0.1 mg
Streptavidin (SA)	60	4 and 1	33 pmoles	~16.7 pmoles (assuming adsorbed form reduces no. of binding sites by 50%)	1	$17.5
Nitrocellulose-binding streptavidin mutant (NC-SA)	24	1 and 0.625	21 pmoles	~20.8 pmoles with biotin-binding domain oriented away from NC-binding domain	<1	$150
Polystreptavidin (pSA)	2000	≤132 and ≤1	Similar to SA	~10.3 pmoles (20 μg binds 414 pmoles biotin from manufacturer)	Similar to SA	$11
Anti-biotin antibody (Ab)	150	2 and 0.2	6.7 pmoles	~2.1 pmoles (assuming factor of $\frac{n}{\pi }$ orientation dependence for n binding sites per molecule)	<<1	$91

## Data Availability

The data presented in this study are available on request from the corresponding author.

## Supplementary Materials

The following supporting information can be downloaded at: https://www.mdpi.com/article/10.3390/mi14101936/s1. Figure S1. pSA binding to biotinylated DNA. (**A**) Representative images of DNA-stained strips after applying premixed pSA at 0.5 mg/mL and biotinylated DNA at various concentrations on nitrocellulose and either rinsing or not rinsing excess protein and DNA. (**B**) Plot of background-correct signal intensity for total DNA deposited (blue “no rinse” condition) and bound DNA to pSA to nitrocellulose (red “after rinse” condition). Data points represent the average of four replicates and the error bars represent the standard deviation. Figure S2. Image data of target DNA hybridization using pSA and NC-SA as protein anchors (three replicates). The results mirror those of the protein and DNA stain data that indicate the pSA anchor system outperforms the NC-SA anchor system under the conditions investigated.
