# Supplementary material for: Protein-Based Anchoring Methods for Nucleic Acid Detection in Lateral Flow Format Assays
Source: Micromachines (Basel). 2023 Oct 16;14(10):1936. doi: 10.3390/mi14101936 (PMC10608873; doi:10.3390/mi14101936)
Supplement: Supplementary file 1 [file micromachines-14-01936-s001.zip › micromachines-2642768-supplementary.pdf]

micromachines

Supplementary Information

Protein-based anchoring methods for nucleic acid detection in lateral flow format assays

Kira Hallerbach[a†], Khadijeh Khederlou[a†], Lael Wentland[a], Lana Senten[a], Steven Brentano[b1], Brian Keefe[b2] and Elain Fu[a*]

[a]School of Chemical, Biological, and Environmental Engineering, Oregon State University, Corvallis, OR 97331
[b1]HP Inc., Palo Alto, CA, [b2]HP Inc., San Diego, CA
* Correspondence: author: elain.fu@oregonstate.edu
†Co-first authors

1. Protein pSA to biotinylated DNA ratio for which the biotinylated DNA saturates pSA

The premix contained 0.5 mg/mL of pSA and varying concentrations of biotinylated DNA from 0 to 80 µM. A volume of 0.5 µL of the capture reagents was hand spotted onto the nitrocellulose membrane and the membrane was dried overnight. One set of membranes was rinsed to remove excess protein and biotinylated DNA, while another set was not rinsed. Both sets of membranes were stained with methylene blue for DNA visualization. The image data is shown in **Figure S1A**, while the background-corrected signal is shown in **Figure S1B**.

A

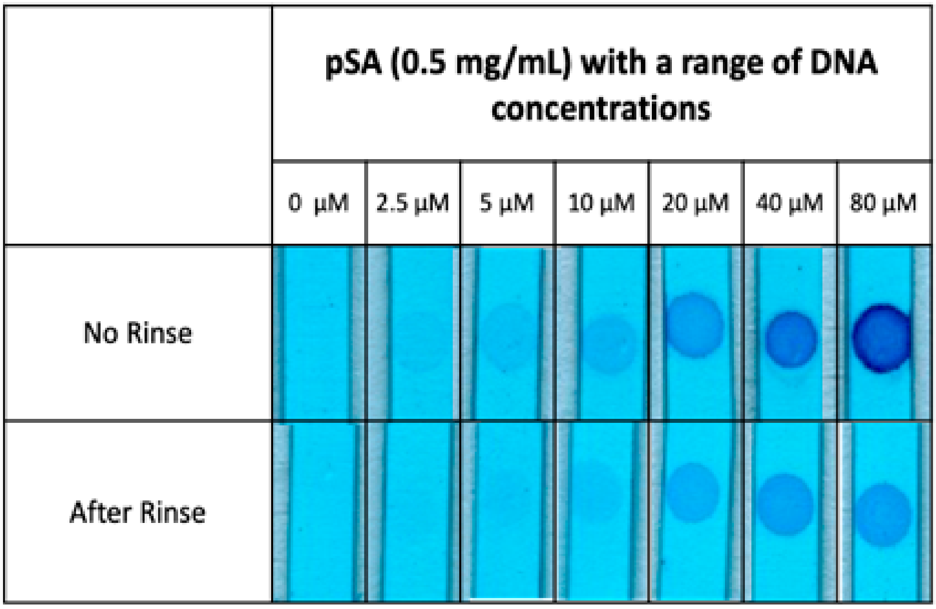

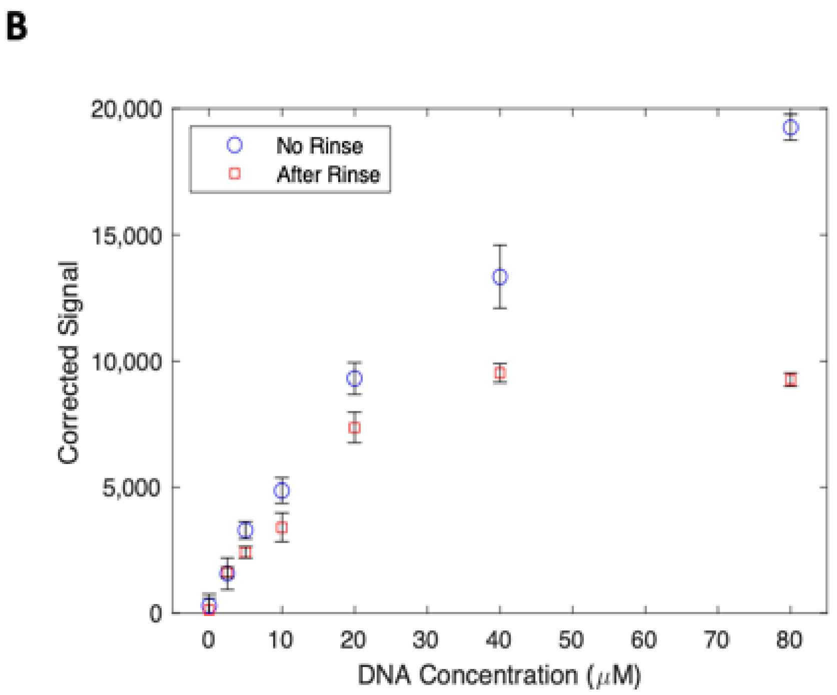

Figure S1. pSA binding to biotinylated DNA. (A) Representative images of DNA-stained strips after applying premixed pSA at 0.5 mg/mL and biotinylated DNA at various concentrations on nitrocellulose and either rinsing or not rinsing excess protein and DNA. (B) Plot of background-correct signal intensity for total DNA deposited (blue "no rinse" condition) and bound DNA to pSA to nitrocellulose (red "after rinse" condition). Data points represent the average of four replicates and the error bars represent the standard deviation.

The "no rinse" signal represents DNA that is attached to the nitrocellulose via pSA adsorption and DNA that is not attached to the nitrocellulose, i.e., all the DNA in the premix that was deposited on the membrane (assuming the staining process is minimally invasive). The "no rinse" signal increases approximately linearly with DNA in the concentration range investigated. The "after rinse signal" represents DNA that is attached to the nitrocellulose via pSA adsorption. (In independent experiments, deposition of DNA only to the nitrocellulose followed by rinsing, effectively removes DNA to a level that is not detectable by methylene blue staining.) The "after rinse" signal increases approximately linear, but then rolls over around 30 to 40 μM biotinylated DNA. This data indicates that a concentration of 0.5 mg/mL of pSA in the premix is saturated by approximately 40 μM of biotinylated DNA. For the pSA experiments in the article, this concentration ratio guided our premix protocols.

2. Target DNA hybridization for pSA vs. NC-SA with biotinylated capture DNA

For completeness, we include the image data for target DNA hybridization associated with the comparison of pSA and NC-SA anchor strategies in **Figure 4**, as **Figure S2**. Following preparation of the capture reagents in each case, an additional set of striped membranes were investigated for target DNA binding at 50°C. Solutions (15 μL) of fluorescently labeled target DNA in PCR buffer, 0.5 μM, 0.25 μM, and 0 μM, were applied to glass fiber sample pads at the upstream edge of the nitrocellulose and allowed to flow for 2 minutes. A final rinse with 30 μL of PCR buffer with 0.1% Tween 20 was applied to the glass fiber pads (in two applications of 15 μL), and then the strips were dried for 1 hour in a covered desiccator. The strips were imaged under a blue light illuminator. The image data shows substantial fluorescence signal for the pSA and biotinylated DNA striped

membranes and no significant fluorescence signal for the NC-SA and biotinylated DNA striped membranes.

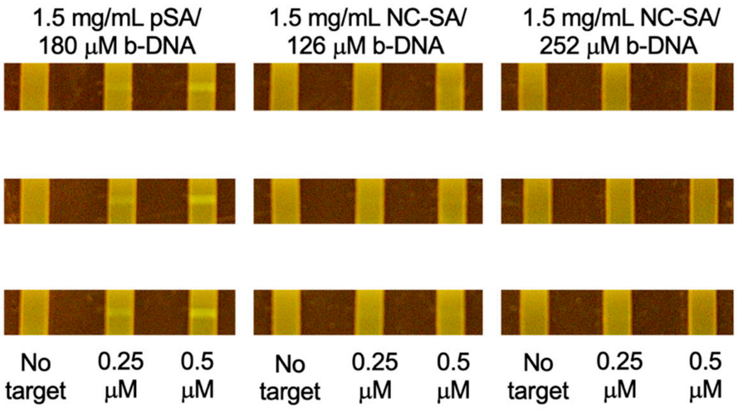

Figure S2. Image data of target DNA hybridization using pSA and NC-SA as protein anchors (three replicates). The results mirror those of the protein and DNA stain data that indicate the pSA anchor system outperforms the NC-SA anchor system under the conditions investigated.
